# Author Correction: Time-restricted feeding restores muscle function in *Drosophila* models of obesity and circadian-rhythm disruption

**DOI:** 10.1038/s41467-020-16193-w

**Published:** 2020-05-15

**Authors:** Jesús E. Villanueva, Christopher Livelo, Adriana S. Trujillo, Sahaana Chandran, Brendon Woodworth, Leo Andrade, Hiep D. Le, Uri Manor, Satchidananda Panda, Girish C. Melkani

**Affiliations:** 1 Department of Biology, Molecular Biology Institute and Heart Institute, San Diego State University, San Diego, CA 92182 USA; 2 Waitt Advanced Biophotonics Center, Salk Institute for Biological Studies, La Jolla, CA 92037 USA; 3 Regulatory Biology Laboratory, Salk Institute for Biological Studies, La Jolla, CA 92037 USA

**Keywords:** Circadian rhythms, Circadian rhythms, Cytoskeleton, Cytoskeleton, Mechanisms of disease

Correction to: *Nature Communications* 10.1038/s41467-019-10563-9, published online 20 June 2019

The original version of this Article contained errors in confocal images in Fig. 3, Fig. 4, and Supplementary Fig. 3. During the first and second resubmissions of this article, the images in question were accidentally improperly named when imported to Power Point for figure preparation which led to several duplications.

The following figure panels were inadvertently and erroneously duplicated: Figure 1h panel “WT-HFD” was duplicated in Fig. 3c as panel “Fln>Sk2KD, 3 W”; Fig. 8h panel “WT TRF-LL” was duplicated in Fig. 3c as panel “Fln/+, 3 W”; Fig. 8h panel “ALF-LL WT” was duplicated in Fig. 4c as panel “Bmm/+, 3 W”; Fig. 6e panel “TRF WT-RD” was duplicated in Fig. 4c as panel “CS control, 3 W”.

Panel “Fln/+, 3 W” in Fig. 3c and panel “WT + bromoenol lactone” in Supplementary Fig. 3a were identical and incorrect.

Furthermore, the Y-axis label in Fig. 3b was absent and should have stated “Relative NLaz Expression”. This has now been added.

The correct version of Fig. 3 is:



which replaces the previous incorrect version:



The correct version of Fig. 4 is:



which replaces the previous incorrect version:



This has now been corrected in both the PDF and HTML versions of the Article.

In addition, the following duplicated panels have the same genotypes but have been replaced by other representative images for clarity: Fig. 6e and Supplementary Fig. 3a panel “WT-HFD ALF”; the image was replaced in Supplementary Fig. 3a. Figure 1h and Supplementary Fig. 3a panel “Sk2”; the image was replaced in Supplementary Fig. 3a.

The errors have now been fixed and the Supplementary Information PDF is available to download from the HTML version of the Article.

All original data for the images presented in these figures can be accessed using the Figshare link https://figshare.com/s/682ff1ae4cde2f2729a4. Additional images from independent replicates can also be accessed using the Figshare link.

