# Supplementary material for: Time-restricted feeding restores muscle function in Drosophila models of obesity and circadian-rhythm disruption
Source: Nat Commun. 2019 Jun 20;10:2700. doi: 10.1038/s41467-019-10563-9 (PMC6586848; doi:10.1038/s41467-019-10563-9)
Supplement: Supplementary file 1 — Supplementary Information [file 41467_2019_10563_MOESM1_ESM.pdf]

Supplementary Materials for

Time-restricted feeding restores muscle function in *Drosophila* models of obesity and circadian-rhythm disruption

Melkani et al., Correspondence and requests for materials should be addressed to G.C.M. (email: gmelkani@sdsu.edu).

This PDF file includes:

Supplementary Figures 1-5

Supplementary Tables 1-3

Supplementary Methods

Supplementary Figure 1

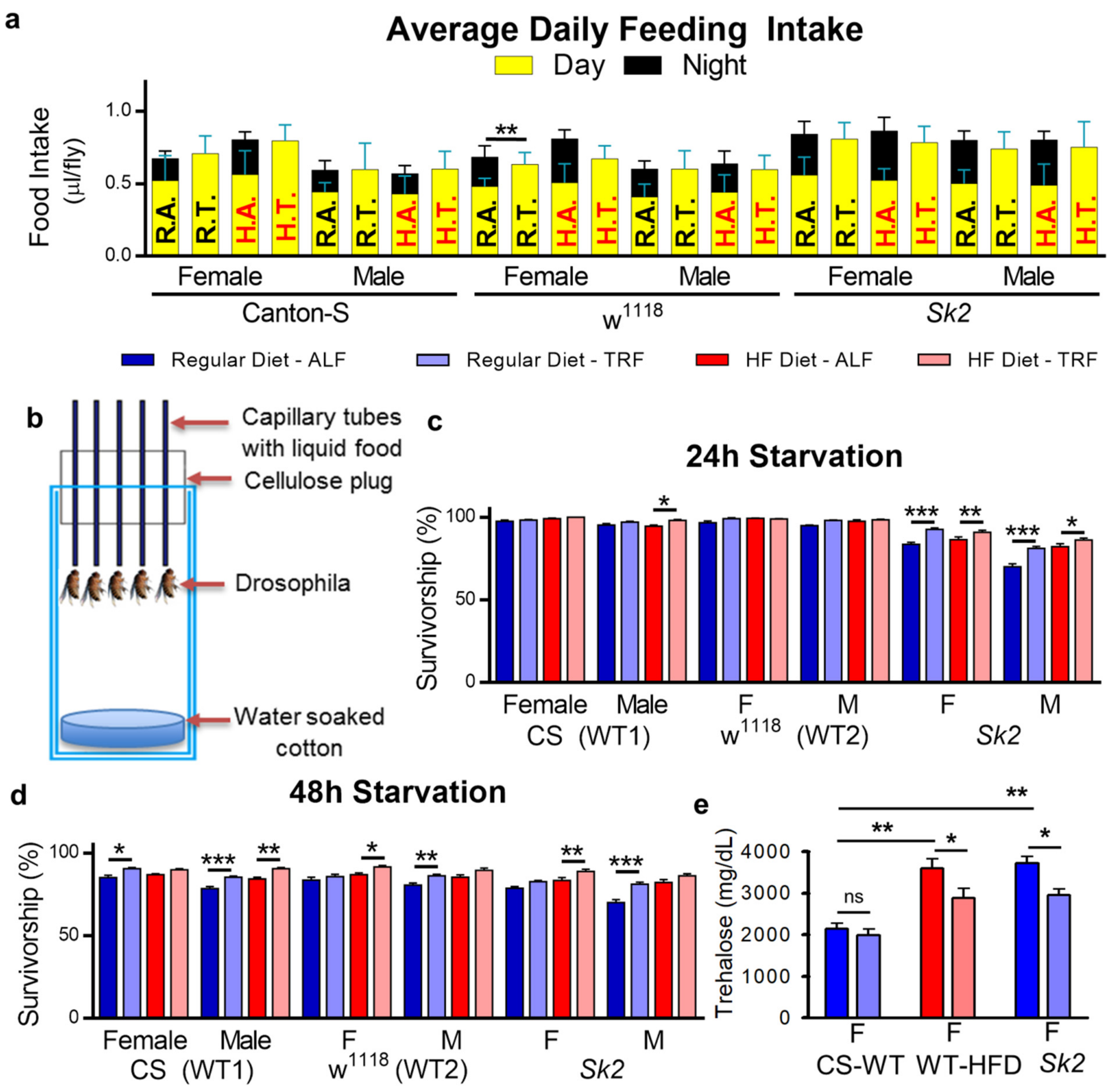

f

Genotype	% of Intact sarcomere (including intact Z-Disk and M-Line)	% of Normal Mitochondria
WT-ALF<br>WT-TRF	96 ± 4<br>97 ± 3^{NS}	94 ± 6<br>95 ± 5^{NS}
WT-HFD-ALF<br>WT-HFD-TRF	84 ± 9*<br>90 ± 7$^{NS/**}$	65 ± 12**<br>88 ±9$^{NS/**}$
Sk2-ALF<br>*Sk2*-TRF	71 ± 8**<br>89 ± 8$^{NS/**}$	51 ± 12***<br>91 ± 6$^{NS/***}$

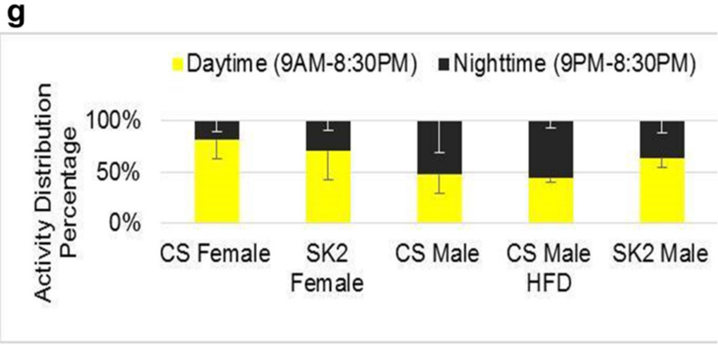

Supplementary Figure 1. Impact of TRF on metabolic parameters. **1a** and **b** CApillary FEeding assay (CAFE). As shown in **b** this assay uses 5 glass capillaries, with liquid food made from 5% (wt/vol), sucrose 5% (wt/vol) autolyzed yeast extract (Bacto yeast extract; BD Diagnostic Systems), and 0.05% dye, held in place by a cellulose plug. Flies are placed in a vial capped with 5 capillaries and 1mL of water in cotton to prevent dehydration. 5 capillaries with food are placed in an empty control vial to account for evaporation of liquid food.

a CAFE Assay Comparisons. Drosophila consumption was measured for 3 consecutive days in cohorts of 10-15 flies and combined to attain an average. Food consumption under TRF was only measured during the 12 hours of food access. N = 50-100, representing 10-12 independent runs. **c** and **d** survivorship after 24- or 48-hours starvation. N = 50, two independent runs. **e** Hemolymph trehalose content under ALF was significantly found elevated in diet and genetically obese flies after 3-weeks compared to age-matched control, which is significantly reduced under TRF in both obesity models but not in the wild-type control. N = 3 independent experiments from extracted Hemolymph from100-150 flies per genotype. **f** Quantification of relative myofibrillar (including Z-disc and M-line integrity and sarcomere architecture including wavy sarcomere), and mitochondrial dysmorphology in TEM images. Obesity resulted in disorganization of Z-discs and M-lines, and mitochondrial dysmorphology, which was significantly improved under TRF. Statistical significance of obesity conditions under ALF were compared with age matched wild-type control (dark blue and dark red ns or asterisks). Statistical significance of HFD and *Sk2* mutant under TRF was compared with age-matched ALF obesity condition and with wild-type TRF (light blue and light red ns or asterisks). **g** Relative activity percentage of 3-weeks old grouped male and grouped female flies (wild-type and obese conditions) from at least 4 days of recording (n=50 each per condition). Data presented for panels a-g as mean ± SD and statistical analysis was performed using one-way ANOVA with post hoc Tukey test, where *= $p<0.05$; **= $p<0.01$***= $p<0.001$; ns = non-significant.

Supplementary Figure 2

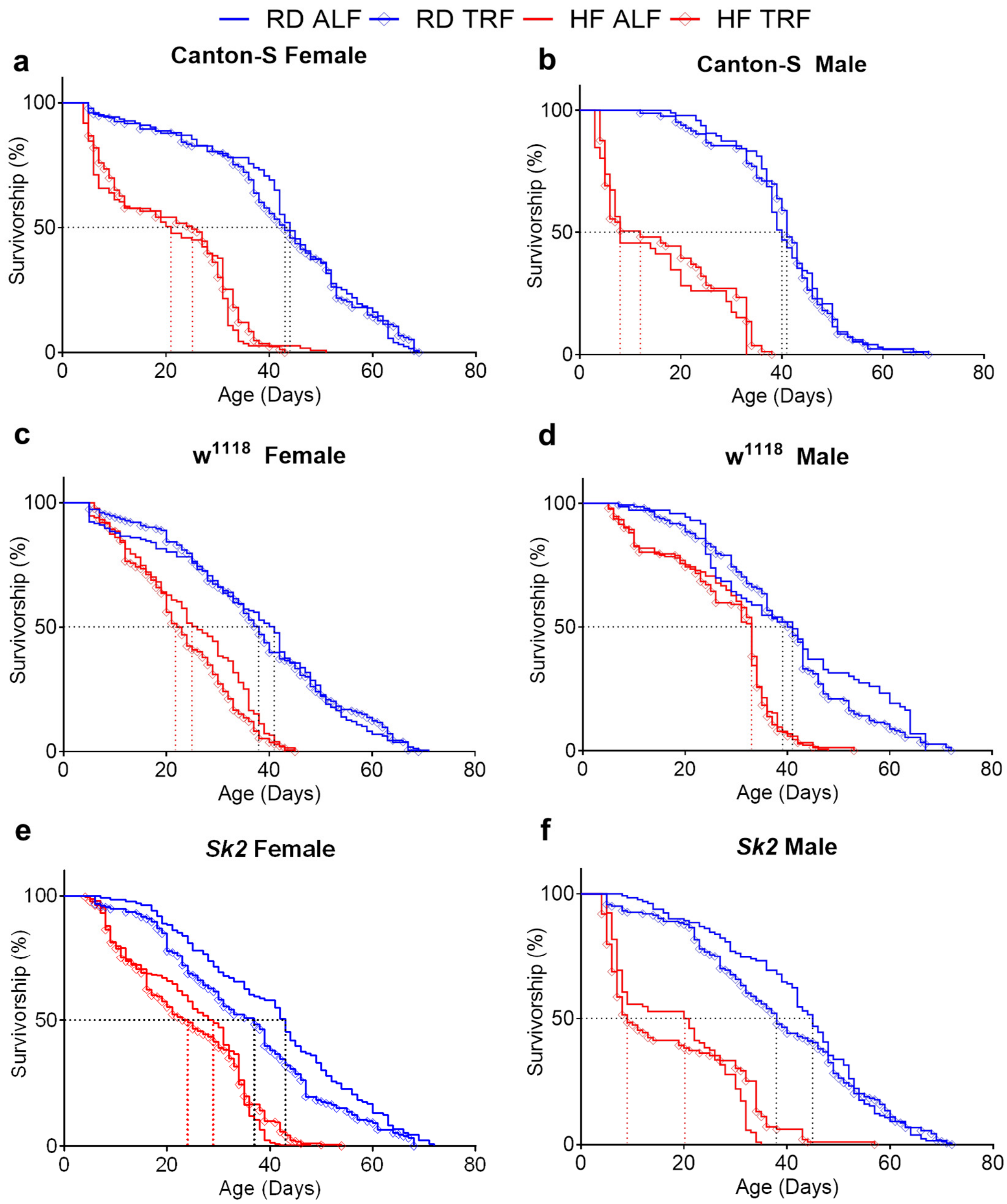

Supplementary Figure 2. Effect of obesity on lifespan and TRF impact. **a-f** Flies were placed into their respective food/feeding regimens after a 4-day developmental period and were kept under constant temperature and humidity under LD (12h Light/Dark). Both diet and genetic obesity resulted in a reduction in lifespan compared to controls. Graphs indicating the percent survival for female adults (N=100 for each group) versus age days post-eclosion. Droplines indicate median survival, where *= p<0.05; **= p<0.01***= p<0.001; ns = non-significant (see Supplementary Tables 1 and 2).

Supplementary Table1. Median Survival. Related to Supplementary Figure 2

Strain (♀/♂)	RD ALF	RD TRF	HF ALF	HF TRF
CS	44/40	43/41	21/8	25/12
w^{1118}	41/41	38/39	26/33	22.5/33
Sk2	43/45	37/38	29/20.5	23.5/9

Supplementary Table2. Statistical Comparisons Related to Supplementary Figure 2

RD ALF vs RD TRF	RD ALF vs HF ALF	HF ALF vs HF TRF
NS/NS	****/****	NS/NS
NS/**	****/****	NS/NS
**/NS	****/****	NS/NS

Supplementary Tables 1 and 2. Medium survival (in days) under ALF and TRF under regular diet (RD) or High fat (HF) diet. Statistical significance among control and flies with obesogenic challenges, as well as between ALF and TRF. Kaplan-Meier used ****p < 0.0001 with statistics calculated using Log-rank (Mantel-Cox) test to determine statistical difference in the lifespan.

Supplementary Table 3: Sequences of primers used in quantitative PCR analyses

Primer Name	Sequence
**Neural Lazarillo (*NLaz*)**	
Nlaz (forward)	GGACAACCCTCGAATGTAACT
Nlaz (reverse)	GACGGCGTATGACTCGTAATC
**Housekeeping gene**	
RPL11 (forward)	CGATCTGGGCATCAAGTACGA
RPL11 (reverse)	TTGCGCTTCCTGTGGTTCAC

Supplementary Table 3. As indicated in the methods section Neural Lazarillo (*NLaz*) expression was carried out using RT-qPCR from micro-dissected indirect flight muscle (IFM). Primers for qPCR are listed in the table.

Supplementary Fig.3

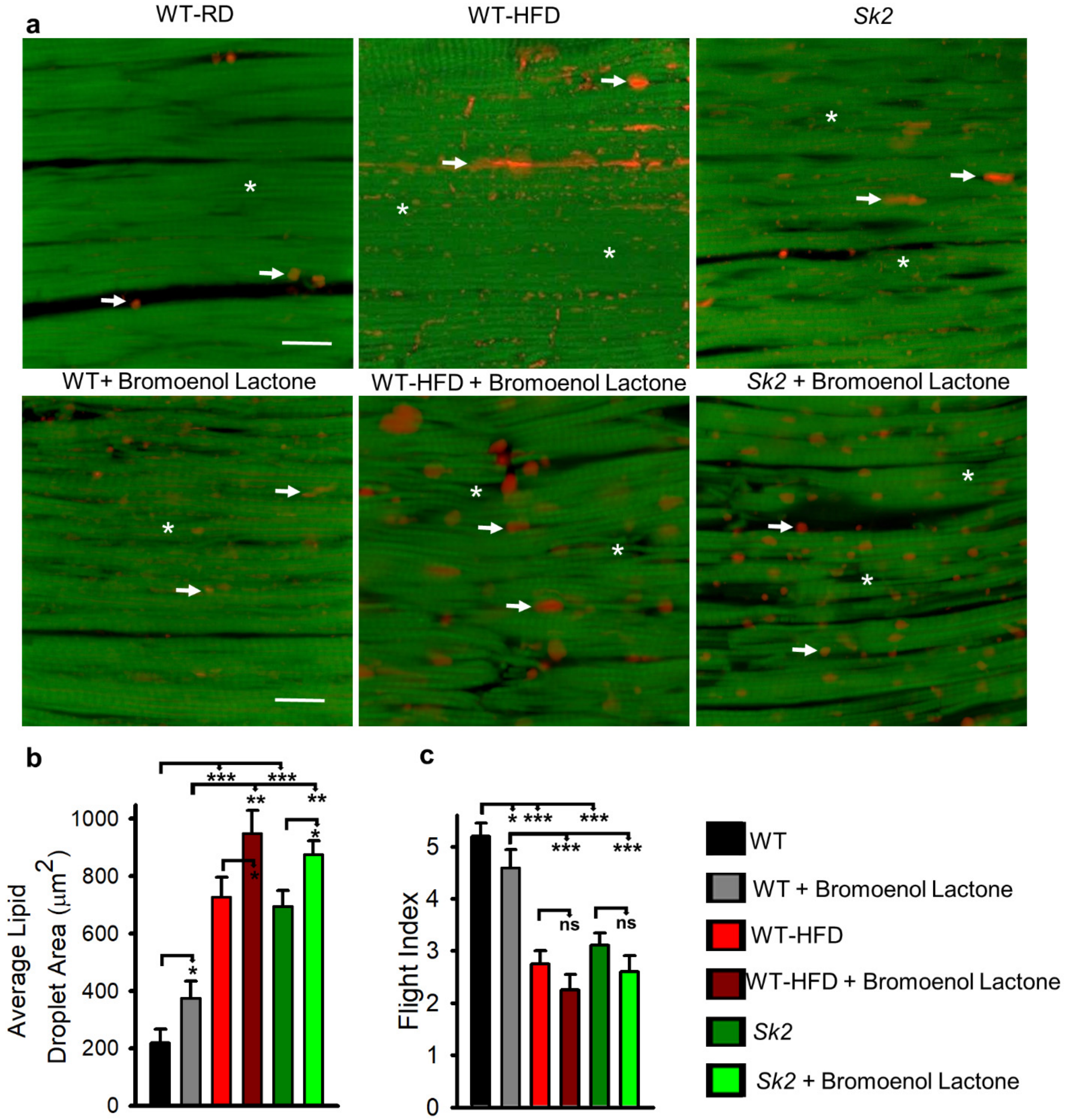

Supplementary Figure 3. Pharmacological inhibition of ATGL (Bmm) impaired muscle function and resulted in enhanced intramuscular lipid infiltration. **a-b** Confocal images of the IFMs from 3-week old females of wild type (WT), HFD and *Sk2* mutant in the absence and presence of BEL probed with phalloidin (green) and Nile Red (red puncta). Scale bar in all images in panel a is 20μm. Dietary supplementation of BEL resulted in more intramuscular lipid (arrows) and actin-containing myofibrillar disorganization in the wild-type and obese background (asterisks), compare to age-matched controls without BEL. **b** Intramuscular lipid area quantification showed significant increase upon supplementation of BEL in all three samples compared to age-matched driver control. N = 9 from three flies IFM in each genotype. **c** Flight performance assay. Fly flight paths were recorded and given a numerical value to calculate flight index (F.I.) (see Methods). Dietary supplementation of BEL resulted in significant reduction in muscle performance in 3-week old male and female

(combined) WT, HFD and *Sk2* mutant flies compared to age matched controls without BEL. (n=100 flies were tested male and female flies under each condition from three independent experiments). Data presented as mean ± SD and statistical analysis was performed using one-way ANOVA with post hoc Tukey test, where *= p<0.05; **= p<0.01***= p<0.001; ns = non-significant.

Supplementary Figure 4

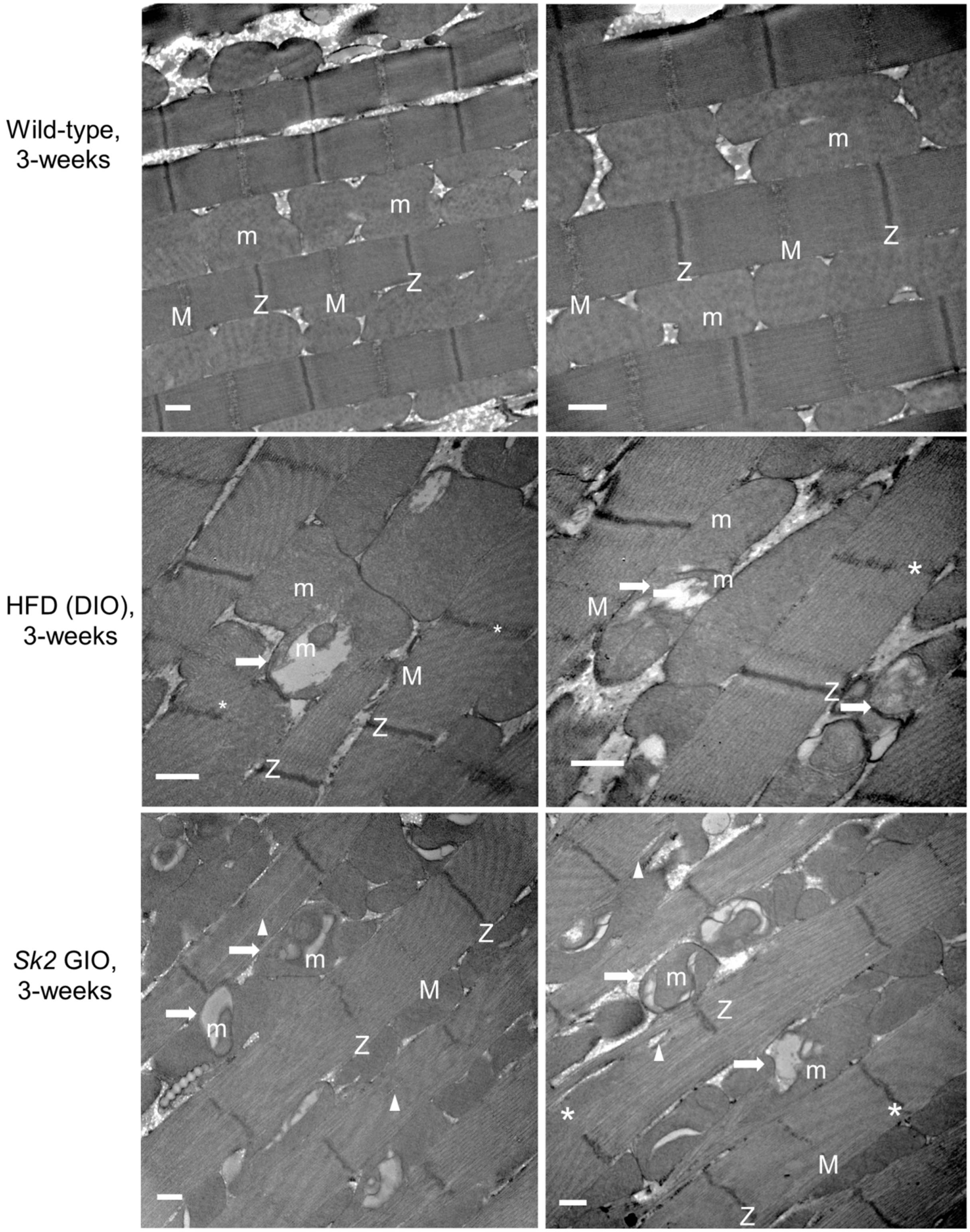

Supplementary Figure 4. Obesity caused abnormal myofibrillar organization and mitochondrial defects.

Ultrastructural analysis using TEM revealed that obesity caused myofibril disorganization and mitochondrial abnormalities compared to wild-type CS under ALF in 3-week old female flies IFM. Compared to control (first two images), occasional myofibrils with partially intact Z-disks (Z) and M-lines (M) were observed (next two images) with the diet -induced obesity which were more prominent in the genetic obesity mutant (last two images). Abnormal mitochondria (m, arrows) and disorganized myofibrils (white arrowheads) were evident with diet-induced obesity. Genetic obesity also resulted in abnormal mitochondria (m, arrows), a more severe myofibril disorganization (white arrowheads) and resulted in wavy sarcomere and Z-disks (asterisks). No such abnormalities were seen in the age-matched control. Scale bar in all six images is 0.5μm. See more description in the manuscript (Figures 2 and 7).

Supplementary Figure 5

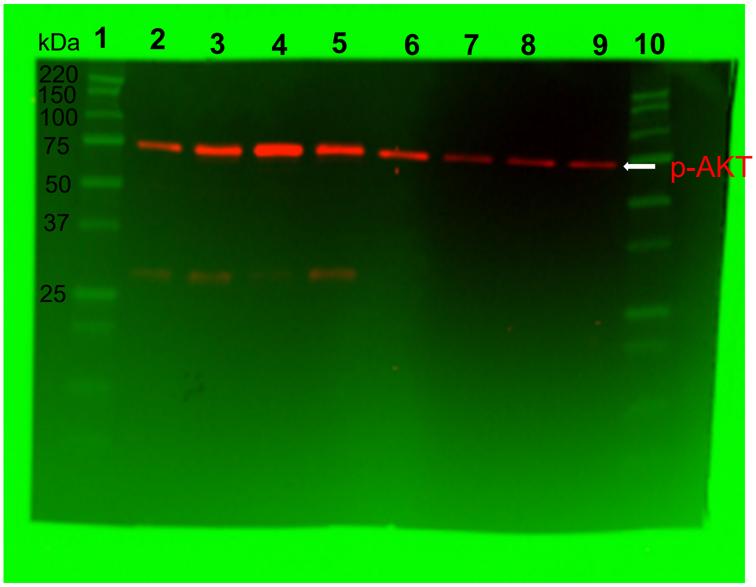

1. Molecular Wt. markers
2. WT-ALF
3. HFD-1-ALF
4. HFD-2-ALF
5. Sk2-1-ALF
6. Sk2-2-ALF
7. WT-TRF
8. HFD-1-TRF
9. Sk2-1-TRF
10. Molecular Wt. markers

Supplementary Fig. 5. Uncropped and unprocessed western blot. Representative uncropped and unprocessed western blot of p-AKT protein levels in IFMs from 3-week-old WT, HFD and *Sk*2 mutant female flies under ALF and TRF.

Supplementary Methods

1. TRF Protocol

Abstract: A 12-hour feeding/fasting paradigm known as Time Restricted Feeding (TRF) is a novel technique shown to help alleviate physiological and metabolic abnormalities associated with genetic and dietary obesity. Here we present a protocol in which shows how *Drosophila melanogaster* (fruit flies) can be put under TRF for the purpose of assessing its protective properties under conditions conferring obesity.

Introduction: Fruit flies can exhibit metabolic disease phenotypes such as sarcomeric disorganization, mitochondrial irregularities, increased skeletal muscle fat infiltration, along with insulin resistance when subjected to obesogenic challenges such as high fat diet and genetic predisposition to obesity.

Time restricted feeding, through the imposition of metabolic homeostasis has shown to slow down aging effects on muscle function, as well as ameliorate phenotypes seen in obesity. After placing fruit flies into TRF conditions, researchers may use downstream assays such as the measurement of flight index, geotaxic index etc to gain insight on metabolic effects of different challenging conditions or change in fruit fly lifestle.

Reagents

Base cornmeal yeast diet

Coconut oil for High Fat Diet (base + 5% oil)

Sucrose for high sugar diet (base + 300 mM)

Combination of both 5% oil and 300 mM sucrose + base for high fat and high sugar diet

Agarose

Equipment

Weighing scale

Autoclave

Fly Vials for pouring in fly food

Cold room for storing fly food

Procedure:

1) Allow new born flies to feed on a regular base diet for the first 3 days after eclosure. This ensures that development of fruit flies are not affected by the challenges.

2) After the 3rd day, separate flies into respective conditions such as high fat diet, high sugar diet, mix of both high fat and high sugar diet for dietary obesogenic challenges and separate genetically predisposed obese flies into regular base diet only.

3) Ensure to keep a consistent schedule of feeding for TRF condition flies and remove flies from fly food and transfer flies into a 1.1% agarose after 12 hours of feeding. After another 12 hours return flies into the same vial of food afterwards. All non-TRF flies remain on fly food for all 24 hours.

4) Continue to follow the same schedule and change fly food vials consistently and uniformly into new vials with new food for both TRF and ALF flies.

5) Perform all assays on fruit flies on 1,3 and 5 weeks of age.

Troubleshooting:

Food seems too sticky and flies get stuck on food surface:

Ensure that you are properly mixing food careful prior to pouring into fly food vials and ensure that you are consistently changing fly food vials no later than 1 week.

Food seems to have bacterial or unnatural growth:

Ensure that you are properly storing all food vials in a cold room to prevent any unwanted bacterial growth.

Time Taken:

5 Weeks of feeding with assay testing at 1, 3 and 5 weeks of age.

3-4 hours for the creation of fly foods

Anticipated Results

Flies under TRF condition are expected to have improved muscle function when compared side-by-side to ALF condition flies. 1 Week of age assay may not show as much significance in improvement compared to longer time points such as 3 and 5 weeks of age.

2. Negative Geotaxis Assay

Abstract: Fruit flies have a natural tendency to oppose the vector of gravity. This negative geotaxis response is taken advantage of in this protocol to test the fruit flies' climbing ability and evaluate muscle performance. Such an assay has also been used by other researchers to test neuronal function associated with the muscle. This assay has also been commonly called the Rapid Iterative Negative Geotaxis (RING) assay.

Introduction: Muscular dysfunction associated with obesity can be apparently observed in the fruit fly model. By utilizing the negative geotaxis assay, researchers can demonstrate severity of muscle impairment by measuring a geotaxic index associated with a group or sample of fruit flies.

In the geotaxis assay, photographs are taken of fruit flies subsequent to perturbation in which flies rapidly respond by scaling the walls of the container in which they are housed. Usually, such perturbation is imparted by tapping the bottom of the vial and subsequently recording the time it takes for fruit flies to reach a threshold distance.

The percentage of the fruit flies able to reach such a threshold distance is calculated and the percentage can represent the climbing ability of that batch of flies.

The geotaxis assay provides a sensitive high throughput assay capable of providing scientists an insight on how locomotor function is affected by certain changes in lifestyle or drug uptake.

Reagents:

Equipment:

Fruit Fly Vial (No Food to keep heights standard)

Background for measuring distance (in cm)

Timer

Camera

Procedure:

1) Carefully mark an empty fly vial with the corresponding group or sample name

2) Measure a 7.0 cm threshold mark on your background to be used alongside your vial

3) House 10-15 flies in labelled empty fly vials

4) Take fly vial containing fruit flies and tap 3 times and immediately begin a 10 second timer.

5)Take a picture of the fly vial alongside your measured background after the 10 seconds is over and repeat 2 more times.

6) Record the number of flies able to reach the threshold and record percentage by taking # of flies able to reach threshold divided by the total amount of flies

7) Finally average the 3 percentages and record or analyze using your desired computer software

Troubleshooting:

Flies are not able to climb up after tapping:

Ensure that you are not tapping excessively hard and that you are using the minimum amount of force to bring all flies to the bottom of the vial prior to recording.

Flies are flying to the top rather than climbing:

Feel free to clip the wings of the flies if flight index will not be used as another assay.

Time Taken:

5-10 minutes for each geotaxis assay/vial

Anticipated Results:

Flies suffering from obesogenic challenges will yield lower percentages of total flies able to climb up the 7.0 cm threshold when compared to TRF or wildtype flies. Other conditions affecting locomotor function will also result in a lower geotaxis index/percentage.

3. Flight Index Assay

Abstract: Measurement of flight performance in *Drosophila melanogaster* is routinely used as a metric of measuring muscle ability and/or muscle defect. We use this technique of measuring flight index to measure the impairment of muscle function upon imposing both dietary (high fat, high sugar or both high fat and high sugar) and genetic obesogenic challenges.

We used fly strains Canton-S and w^{1118} for wild type strains and *Sphingosine kinase 2* (*Sk2,* Bl#14133), Infertile crescent (*Ifc*, Bl#1549) and Brummer (*Bmm,* Bl #15828) for genetically obese strains obtained from Bloomington stocks.

This assay tests the indirect flight muscle (IFM) of the fruit fly which has been implicated to share similarities to the human skeletal muscle. This simple, yet effective assay allows for the quick measurement of the fly's IFM and allows the scientist to gain insight on the fly's muscle function upon mutation or change in lifestyle of the fly.

Equipment:

Plexiglas Box

Light source

Fly Vial

Counter (preferably with 4 categories, 1 for up, horizontal, downwards and no flight)

Modified vacuum system for collecting flies

Procedure

1) Allow flies to feed on respective dietary conditions such as high fat diet for dietary obese flies, regular cornmeal diet for wild type flies and regular cornmeal diet for genetic obese flies. At intervals of 1, 3 and 5 weeks of age, test the flight index of the flies to gain insight on IFM functionality of each category.
2) Initially place a light source (regular light is enough) at the top of the Plexiglas and turn on. Slowly open the vial which houses the desired population of flies for testing and slowly create an opening to release flies one at a time.
3) After the release of each fly, record the trajectory of flight on the counter and repeat until all flies have been released from the vial. After recording all of the flight trajectories for a population of flies calculate the flight index by counting the number of flies in each category and multiply by their point value corresponding to their trajectory, for example 6 flies with upward flight would be multiplied by 6 yielding ((6 flies *6 upward)/6 total flies) a flight index of 6.
4) Collect all flies by using a vacuum which allows flies to be gently placed back into original vial and regrow until the next age interval or dispose if already finished with all data collection.
5) Record all your data and plot using either SigmaPlot or Graphpad Prism

Timing

Fly Aging: 5 Weeks

Flight Testing: 20-30 minutes

Recollection of flies into tube: 5 minutes:

Data Analysis: 2-3 Hours

Anticipated Results:

Flies challenged with obesogenic conditions will display flight impairment and will yield lower flight indices when compared to their wild type counter parts. The significance of this difference between wildtype and obese flies flight index can be further extrapolated using programs such as SigmaPlot or GraphPad Prism through running of statistical tests.
